# Mushroom Detection and Three Dimensional Pose Estimation from Multi-View Point Clouds

**DOI:** 10.3390/s23073576

**Published:** 2023-03-29

**Authors:** George Retsinas, Niki Efthymiou, Dafni Anagnostopoulou, Petros Maragos

**Affiliations:** School of Electrical and Computer Engineering, National Technical University of Athens, 15773 Athens, Greece

**Keywords:** agricultural applications, mushroom detection, 3D pose estimation, instance segmentation, template matching

## Abstract

Agricultural robotics is an up and coming field which deals with the development of robotic systems able to tackle a multitude of agricultural tasks efficiently. The case of interest, in this work, is mushroom collection in industrial mushroom farms. Developing such a robot, able to select and out-root a mushroom, requires delicate actions that can only be conducted if a well-performing perception module exists. Specifically, one should accurately detect the 3D pose of a mushroom in order to facilitate the smooth operation of the robotic system. In this work, we develop a vision module for 3D pose estimation of mushrooms from multi-view point clouds using multiple RealSense active–stereo cameras. The main challenge is the lack of annotation data, since 3D annotation is practically infeasible on a large scale. To address this, we developed a novel pipeline for mushroom instance segmentation and template matching, where a 3D model of a mushroom is the only data available. We evaluated, quantitatively, our approach over a synthetic dataset of mushroom scenes, and we, further, validated, qualitatively, the effectiveness of our method over a set of real data, collected by different vision settings.

## 1. Introduction

Agricultural robotics [1] has emerged as a promising solution to address various challenges faced by traditional farming techniques, such as labor shortages, crop yield optimization, and environmental sustainability. The integration of robotics and automation technologies in agriculture has been shown to increase efficiency, reduce labor costs, and enhance crop quality. The application of robotics in agriculture includes tasks such as seeding [2], planting, fertilizing, and harvesting [3], which can be performed with greater precision, speed, and consistency than is possible with manual labor. Furthermore, the use of robots in agriculture has the potential to improve working conditions for farm workers and minimize their exposure to hazardous environments.

Typical vision systems can provide the necessary information for automated robotic systems. Widely-used modules in such systems include image segmentation (separate regions of interest; for example, different types of plantation), object detection (e.g., fruits), and pose estimation (3D structure of objects of interest). Specifically, pose estimation, which is the task at hand, involves estimating the position and orientation of an object in 3D space relative to the sensor. This latter task is challenging when considering a monocular RGB as the only perception sensor. To this end, we focused on solutions where depth sensors can provide the necessary 3D information (at least partially). With the availability of low-cost 3D sensors, such as LiDAR and stereo cameras [4], the collection of point cloud data has become a more common practice in precision agriculture.

In our use case, we explore the capabilities of robotic automation for mushroom picking in mushroom farms from the vision system perspective. Specifically, a robotic arm should be aware of the mushroom’s location and the whole 3D pose placed suitably to gather it. Since mushrooms grow in clusters, robust computer vision algorithms should be implemented into agricultural robots for the robots to recognize mushrooms and separate them from the remainder of the cluster. As far as we know, the topic of mushroom recognition and segmentation is primarily addressed as a two-dimensional image detection problem. Such methods may be useful for a vision system that monitors mushroom growth, but are insufficient for a robotic harvester. Due to the scarcity of research on mushrooms’ 3D posture estimation, mostly justified by the scarcity of 3D annotated data, only the approach of Baisa et al. [5] addresses the same task. In more detail, the approach of [5] is based on a detection step from an overhead camera, using a circle detection method, and estimates the mushroom pose using a standard off-the-shelf registration pipeline over point cloud data. Nonetheless, such registration algorithms (RANdom SAmple Consensus algorithm for global registration and Iterative Closest Point algorithm for finer rigid registration) have proven to be very sensitive and prone to erroneous estimations, while segmentation of clustered mushrooms can be very challenging, especially from an overhead view. To this end, we propose several modifications to address common issues of standard detection and registration pipelines in the context of mushroom picking applications.

In this work, we aimed to simultaneously tackle the detection and pose estimation of mushrooms using point clouds of mushroom scenes as input. To provide a precise recreation of the 3D scene, we considered multi-view approaches of multiple RGB-depth sensors. The main challenge we faced in the pose estimation task was the lack of 3D annotation. In fact, 3D annotation of the pose is practically infeasible on a large scale. To this end, we developed an annotation-free template-based approach that relies on prior knowledge of the 3D shape of a mushroom in the form of a simple 3D template model. In a nutshell, the contributions of this work are summarized as follows:

- Explore multi-view vision systems for geometry-preserving point clouds.
- Explore RANSAC-based (RANdom SAmple Consensus [6]) solutions for template matching.
- Propose a two-step pipeline for detecting mushrooms and estimating their poses. The first step is a novel instance-segmentation approach, while the second introduces a modified version of the Iterative Closest Point algorithm for template matching. The whole pipeline is learning-free, in the sense that no training based on annotation was involved.
- Introduce an ellipsoid-based lightweight alternative for pose estimation, relying on the resemblance of the mushroom cap shape to ellipsoids.
- Develop a pipeline for creating synthetic mushroom point cloud scenes that approximate realistic scenes. This pipeline was used for creating a validation dataset for quantitative evaluation of the proposed approach.

The remainder of this paper is organized as follows: Section 2 presents previous works in computer vision algorithms for mushroom-related applications. Section 3 presents the main goal and limitations of a 3D pose estimation of mushrooms from multi-view point clouds and the employed setup. Section 4 outlines the phases of a point cloud preprocessing pipeline, while in Section 5, we describe our initial algorithm for pose estimation via RANSAC-based template matching. Section 6 explains our two-step proposed algorithm for mushroom detection and 3D pose estimation. Section 7 includes our experimental evaluation, both on a synthetic dataset and real data, and Section 8 concludes the work.

## 2. Related Work

It is inevitable that agriculture, as with most of the productive sectors, has altered a lot because of technological achievements, such as the following: advanced wrist detection systems [7], sophisticated weed spraying machines [8], and robots for crop harvesting [9]. Wosner et al. [10] discussed the application of modern deep networks in visual object detection for automated agriculture, benchmarking their performance on seven diverse datasets and comparing their accuracy to human performance. The results suggested that a multiple-resolution approach is necessary for handling small objects and large scale variance. The paper also suggests application-specific accuracy metrics for estimating the best performing detectors.

Lately, there has been an increasing interest in computer vision applications for mushroom cultivation. Specifically, in [11], the authors reviewed papers published between 1991 and 2021 on computer vision and machine learning topics applied in the mushroom industry and came to conclusions on the limitations and future prospects. Hyperspectral imaging was used by both [12,13] in order to predict mushroom moisture content and damage detection correspondingly. On the other hand, in [14], the authors employed digital images of two-dimensional correlation spectroscopy for the species discrimination of bolete mushrooms with residual neural networks.

Closer to the task at hand, there are several works concerning mushroom detection from 2D images, treating this task as a typical object detection paradigm. The authors in [15] examined traditional image processing methods for mushroom detection, such as thresholding, morphological techniques, and segmentation metaphors in real-world examples, and showed that they could not fully meet the demands of morphological diversity. Most of the proposed methods are based on 2D RGB images concerning mushroom detection and positioning. In [16], the authors proposed an algorithm, based on convolutional neural networks (YOLOv3), to estimate the bounding box locations of each mushroom using images continuously captured by a camera. A score–punishment algorithm (that sequentially applied color quantization, side object removal, contour detection, central point search, and fine-tuning) was applied in order to calculate the circle diameter of round mushroom caps. Ref. [17] proposed a deep learning algorithm called Mushroom-YOLO for shiitake growth recognition and showed the effectiveness of the proposed approach and its potential for controlling the quality of shiitake mushroom growth without human intervention in indoor farming. Furthermore, Ref. [18] proposed a new object detection algorithm called Recursive-YOLOv5, which can detect edible mushrooms in real-time using industrial cameras and can be applied to automatic picking machines. Recursive-YOLOv5 introduces several improvements to the YOLOv5 network, including recursion, atrous spatial pyramid pooling, and distance IoU non-maximum suppression.

Addressing works dealing with similar tasks, but on different crops, Ciarfuglia et al. [19] tackled the difficulty of generating pseudo-labels for agricultural robotics applications due to the scarcity of annotated data for fruits in orchards. Thus, they presented a method for producing high-quality pseudo-labels by combining self-supervised and transfer learning. On the other hand, Fei et al. [20], in order to enable more data-efficient and generalizable neural network models in agriculture, proposed a method that generates photorealistic agricultural images using a semantically constrained Generative Adversarial Network (GAN) to preserve fruit position and geometry. The method can significantly speed up the domain adaption process, increase performance, and decrease labeling requirements.

Concerning segmentation task, a CNN-based technique for segmenting and recognizing broccoli heads from 3D point clouds was provided by Le Louedec et al. [21]. They tested their method on broccoli fields by examining both semantic and instance segmentation and offered a qualitative analysis. Wang et al., in [22], worked on instance segmentation in 3D point clouds. They used point clouds from real and artificial datasets to train PartNet, using a point cloud of lettuce as input and extracting the segmentation of each leaf as output. In [23], the authors presented the LeafMask neural network that uses an anchor-free instance segmentation paradigm to accurately count and segment the leaves of plants for high-throughput plant phenotype data analysis.

As far as we know, research on mushrooms’ 3D posture estimation is limited. Qian et al. [24] proposed an object detection and localization approach for an oyster mushroom-picking robot that combines detection information from a neural network with depth information from an RGB-D camera. This approach used the SSD object detection algorithm and depth images, based on binocular and structured light principles, to locate the precise position of the detected object in the 3D environment, guaranteeing real-time performance. In [5], which has the most similar approach to our work, the authors describe a method for detecting and segmenting mushrooms in 3D space using RGB and depth information. The mushrooms are detected using a combination of active contouring and circular Hough transform, and their 3D positions and orientations are estimated using a template mushroom model via registration techniques.

## 3. Problem Statement: Vision Setup and Limitations

Since the main goal was the 3D pose estimation of mushrooms, an approach that relies only on single-view RGB images is almost infeasible, especially given the lack of annotated data. We investigated the possibility of achieving 3D understanding based on single-viewed RGB scenes, but this task proved to be extremely challenging.

The easiest and most efficient way to address the problem of pose estimation, a 3D problem by its nature, is to obtain a mushroom scene in a form that closely resembles its 3D structure. Implementation-wise, this can be effectively achieved by obtaining point clouds using depth cameras from multiple views. To this end, we considered using depth, along with RGB information, via an RGB-D camera sensor. This extra depth information allowed the input data to be formatted into 3D point clouds, approximating the captured 3D structure. However, a single view RGB-D might still not provide all the necessary information for the task of 3D pose estimation, especially when considering occluded mushrooms from a specific view. Such partial structural information could lead to erroneous estimations. To address this issue, we considered vision setups where multiple RGB-D sensors are used in order to obtain point clouds from different views.

**Figure 1:**
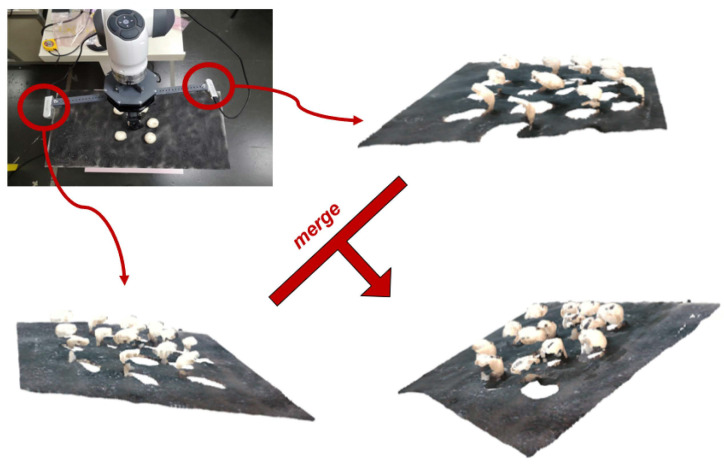
Indicative vision setup for obtaining point clouds from different views. Two RealSense depth cameras are used, with a rotation option of the cameras around *z*-axis. In the simplest scenario, two pointclouds, one from each camera, are collected and merged into a single point cloud of the scene.

All considered vision setups rely on Intel^®^ RealSense™ depth cameras (D435 version), which rely on active stereo technology for depth estimation. An indicative setup is depicted in Figure 1, where two RealSense cameras are mounted on a 3D printed extension that can be rotated (along the *z*-axis) in order to explore the ability to enrich the point cloud information under multiple viewpoints. The angle of the cameras and their distance can be modified.

**Figure 2:**
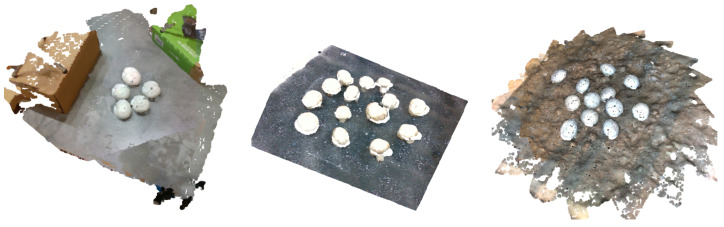
Three different settings of collected validation data: merged point cloud from 3 views using 3D printed mushrooms (**left**), merged point cloud from 2 views using real mushrooms (**center**) and merged point cloud from multiple views (18) using 3D printed mushrooms (**right**).

Specifically, three different setups were considered in practice:

- A proof of concept setup with three RGB-D cameras placed on the perimeter of a circle above the mushroom scene (one camera every 120 degrees). The mushroom scenes explored with this setup were simplistic and contained 3D printed mushrooms, as shown in Figure 2 (left). While developing our methods, we used this setup to validate the significance of each step, providing visual examples throughout this manuscript.
- The setup of Figure 1 was used for collecting point clouds from two opposite cameras, without rotating the cameras. Real mushrooms were used to create the scenes. An example of a point cloud obtained from this setup is shown in Figure 2 (center).
- A similar setup of rotating cameras, developed by our project collaborators in TWI Hellas (THL), was used to create a collection of multi-view point clouds. Our goal was to recreate the actual 3D mushroom scene as accurately as possible, utilizing a rotating vision system that consisted of two cameras that could move along the perimeter of a circle. In order to scan a specific mushroom scene, the vision system captured nine snapshots of the scene. Between each snapshot, the camera system rotated 20°. The RGB-D snapshots from the scanning were converted to point clouds stitched together (a preliminary stitching estimation was performed with an Iterative Closest Point (ICP) registration algorithm, given an initial estimation of the point cloud position from the camera parameters and the camera orientation). Figure 2 (right) shows an example of a point cloud obtained from this setup.

To sum up, the setup with the pair of rotating active stereo cameras produced high quality point clouds and fulfilled all the following above-mentioned requirements: accurate depth estimation given the mushroom features, namely shape, color, and texture; noise elimination; ability to reach all areas of mushroom beds.

**Figure 3:**
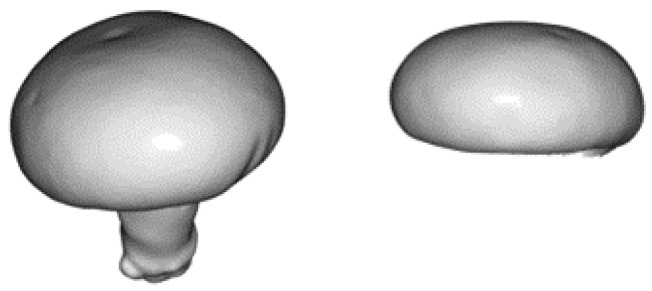
The 3D mesh of the mushroom template. In practice, we used only the 3D model of the mushroom cap (**right**), instead of the whole mushroom mesh (**left**).

Our main problem, as already stated, was the absence of (a sufficiently large amount of) annotated data; thus, we could not train a typical end-to-end deep learning system in order to detect mushrooms and their poses. Our goal was to develop a robust and fast pipeline for detecting mushrooms when the only data available was a single mushroom template in the form of a 3D mesh model, as shown in Figure 3.

## 4. Point Cloud Preprocessing

**Figure 4:**
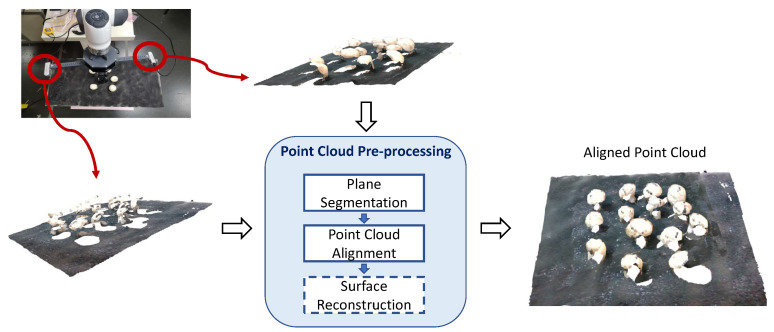
Overview of the preprocessing steps. The input is a set of point clouds from different views. The surface reconstruction step is optional.

Here we briefly describe the necessary steps (plane segmentation and fine-alignment of multiple point clouds), and the optional steps (mesh creation from point clouds), of a point cloud processing pipeline that could be helpful in any of the forthcoming proposed algorithms. An overview of the steps can be found in Figure 4. Visual examples accompany the description of these steps for the case of multi-view input from 3 Intel^®^ RealSense™ cameras.

### 4.1. Plane Segmentation

The problem at hand concerned segmentation and 3D pose estimation of mushrooms grown in mushroom farms. There, mushrooms are cultivated over industrial shelves, essentially defining a reference plane. To this end, the first step is plane segmentation, in the sense of separating the “ground” from the actual object (i.e., mushrooms) in the foreground. This step benefitted us in two distinct ways:

- We removed the background irrelevant to mushroom detection and pose estimation, considerably simplifying the scene and reducing the point cloud size.
- We obtained auxiliary information about the point cloud’s direction in space, namely the direction of the normal of the extracted plane. This could be helpful to quickly initialize the forthcoming point cloud alignment step, when multiple point clouds from different views were available.

**Figure 5:**
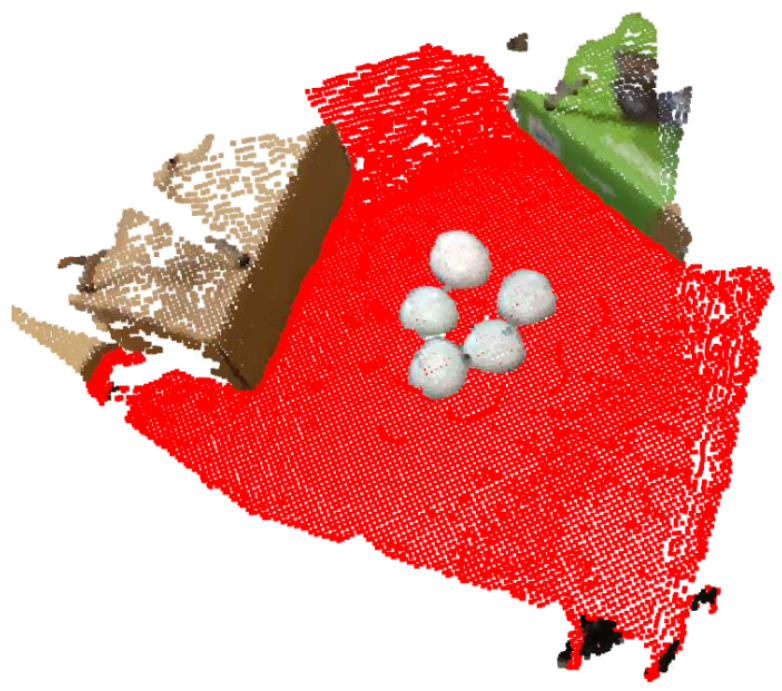
Plane segmentation; points belonging to the detected ground plane are visualized with red color.

Implementation-wise, the plane segmentation step was performed via the RANSAC algorithm, that tries to estimate the best possible plane (using four parameters, i.e., ax+by+cz+d=0) with the most inliers, given a distance threshold. The plane RANSAC algorithm is applied over a down-sampled point cloud with a user-defined voxel size. The distance threshold of the plane segmentation process is controlled by this user-defined voxel-size (×5). This step’s results are visualized in Figure 5, where the detected plane is depicted with red color.

### 4.2. Alignment of Multiple Point Clouds

**Figure 6:**
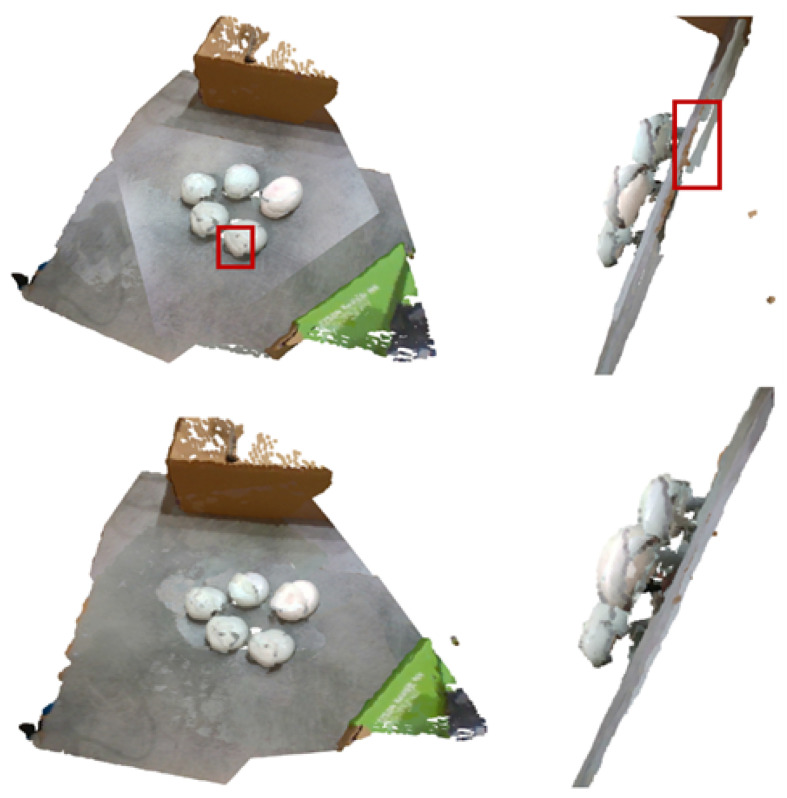
Upper row: Examples of the initial alignment using the camera parameters, bottom row: Examples of the finer alignment of the developed method. Red boxes point out misalignments.

The provided point clouds were initially aligned according to a camera registration algorithm. Even though this initial estimation was good enough, the point clouds were not fully aligned, as depicted in Figure 6. To this end, we utilized the colored point cloud registration of [25]. This method is a modified version of the ICP algorithm, with multi-scaled sub-steps, using different voxel sizes to have a coarse-to-fine registration result. A “good enough” initial estimation of the registration was assumed for this iterative process to be effective. This assumption was in-line with the expected initial registration from the cameras’ extrinsic parameters. Since we had multiple point clouds in some cases, we re-ran this algorithm a few times (in practice, we used 5× the number of initial point clouds). Specifically, for the case of more than two point clouds, each time a random point cloud was selected as the source to be optimized, the remaining point clouds were merged as the target point cloud; thus, iteratively (re-)adjusting a point cloud at each step. The resulting combined and fine-aligned point cloud can be found in Figure 6. Note that the planes are notably better aligned.

#### Mesh Reconstruction

A surface mesh reconstruction from the point cloud was followed as an intermediate, but optional, step to help remove ambiguities that point clouds inherently introduce. In other words, we tried to strictly define the surface under investigation in order to fully understand its characteristics.

To this end, we developed a surface reconstruction algorithm, that shares the core idea of many such algorithms, namely, applying a Delaunay triangulation over patches of the point cloud using a 2D projection. The following steps briefly describe this reconstruction scheme:

- Vertex Estimation: Sample the initial point cloud with a voxel-based downsampling approach. The resulting points constitute the initial estimation of the vertices of the mesh.
- Manifold Embedding: Project the 3D points into a 2D space while retaining the local geometry of the 3D structure. This is a typical case of manifold embedding. We used the ISOMAP (ISOmetric feature MAPping [26]) algorithm. Projecting the whole point cloud has two major disadvantages: (1) if the geometry is complex, local details cannot always be retained, and (2) the computational complexity is high since ISOMAP computes the shortest path (Dijkstra algorithm) and subsequently an eigenvalue decomposition. Both disadvantages can be effectively addressed by breaking the point cloud into segments. This was performed by k-means clustering. In practice, we used a large number of clusters to speed up the procedure (K = 200). In order to track cross-segment regions, we added, to each cluster, a number of neighboring points from other clusters.
- Face Estimation: Given the projected 2D point set for each cluster, perform a Delaunay triangulation. The generated triangles are the faces of the 3D shape. The necessity of this triangulation step was the motivation behind the request for unfolding the 3D manifold into a 2D space.
- Post-processing: Remove triangles with unnaturally long edges in the 3D space (cases of violated local structure into the projected space). Remove duplicate faces that may occur due to the neighboring points in each cluster. Finally, apply Laplacian smoothing to the generated mesh.

**Figure 7:**
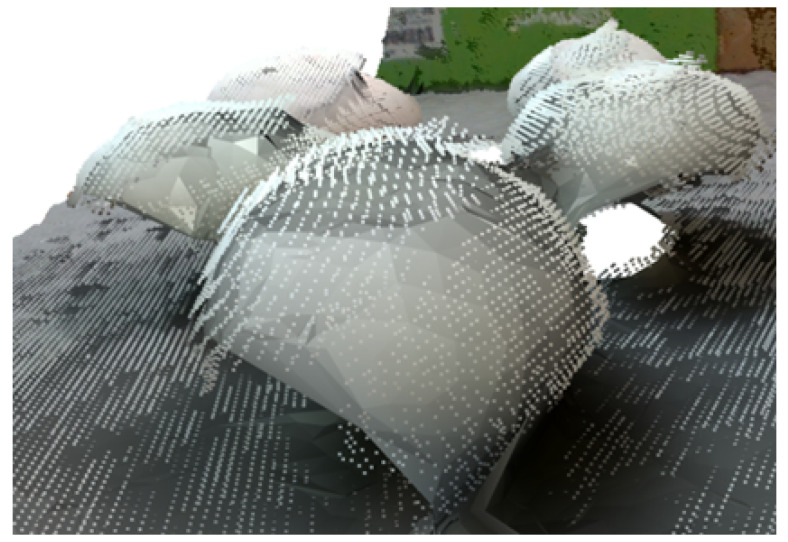
Reconstructed mesh overlaid by the initial point cloud.

**Figure 8:**
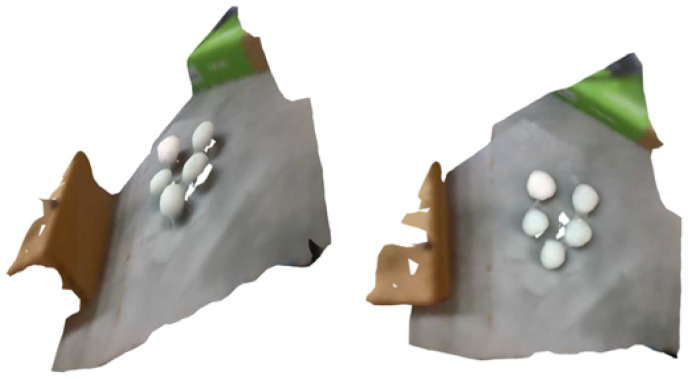
Visualization of the reconstructed mesh for different viewpoints.

A close-up example of the generated, and the corresponding, point cloud can be seen in Figure 7, where it can be observed that the underlying mesh provided a better estimation of the mushroom curvature. The reconstruction of the whole scene is shown in Figure 8.

**Limitations:** Despite the effectiveness of the approach and the reduced complexity of the proposed modification that used k-means, the overall processing time was approximately 10 s for the depicted detailed meshes (25K vertices) and could be reduced to 1–2 s for a more crude mesh of 9K vertices. Such time requirements are not helpful for real-time applications, but this process should not be performed at high frequency. Moreover, the forthcoming algorithms rely on point clouds and not on mesh representations. Therefore, one should sample the reconstructed mesh. To this end, we should explore different and more efficient ways to provide this surface-like effect directly on the point cloud representation without the extra overheads.

## 5. First Attempt: RANSAC-Based Template Matching

Given that we had a mushroom template in a 3D mesh model, we could develop a pose estimation pipeline by following typical template matching strategies. Specifically, in this work, we started by appropriately adapting a typical RANSAC-based template detection pipeline.

Given the nature of the task, template estimation should rely only on the mushroom cap, trying to detect its distinct curvature, rather than include the mushroom’s stem, which is mostly non-visible. To this end, we used, as a template, a mushroom cap, as shown in Figure 3.

### 5.1. The 3D Features

The performance of RANSAC relies heavily on the quality of the extracted correspondences, and, thus, on the quality of the features extracted. To this end, we explored two different 3D features, designed to describe point cloud regions: (1) FPFH features (Fast Point Feature Histograms) [27] and (2) FCGF (Fully Convolutional Geometric Features) [28].

In more detail, the widely-used FPFH descriptors are handcrafted features, which aim to faithfully capture the geometry around a point using a multi-dimensional histogram of values, describing the relationships between all pairs of points in the neighborhood (the term ‘fast’ stems from a variation in the computation of these histograms which leads to faster estimation). This representation is compact (dimensionality of 33) and invariant to the 6D pose of the underlying surface.

The very recent FCGF is a modern alternative, based on a 3D fully-convolutional network, which is further assisted, during training, by the introduction of extra metric learning losses (contrastive loss over “hard” cases). We used a pre-trained network (already trained in 3DMatch dataset [29]) to create compact descriptors of 32 dimensions.

### 5.2. Template Matching Pipeline

The template matching algorithm is briefly described as follows:

- Subsample both template and scene point clouds using a voxel-based downsampling method. This step speeds up the whole procedure.
- Extract 3D features for the downsampled point clouds. Each point corresponds to a feature. Two options are available, FPFH and FCGF features, as mentioned above.
- Perform RANSAC matching: impose strict criteria (e.g., have very similar normals) for validating a set of correspondences. Optionally, we could re-run the RANSAC matching step and select the run with the higher fitness value. This might be helpful due to the relatively large scene and the probabilistic nature of RANSAC.
- Perform fine-tuning of the detected template transform using ICP. To assist the ICP algorithm, we extracted the points from the scene under a specific radius threshold from the transformed points under the detected transform from the RANSAC step. Note that ICP is rather sensitive, and, thus, a good initialization is necessary from the RANSAC step.

This pipeline could detect a single mushroom with close-to-real-time efficiency (∼0.2 s). The final two steps for detecting multiple mushrooms must be repeated after deleting the points corresponding to the detected mushroom. We should highlight that detecting multiple mushrooms from the start is not required for the robotic task at hand. The vision system only needs to correctly identify the pose of a single mushroom to be grasped in the upcoming robotic grasping/uprooting step. Detecting other mushrooms can be done in parallel with the robotic functionalities.

**Figure 9:**
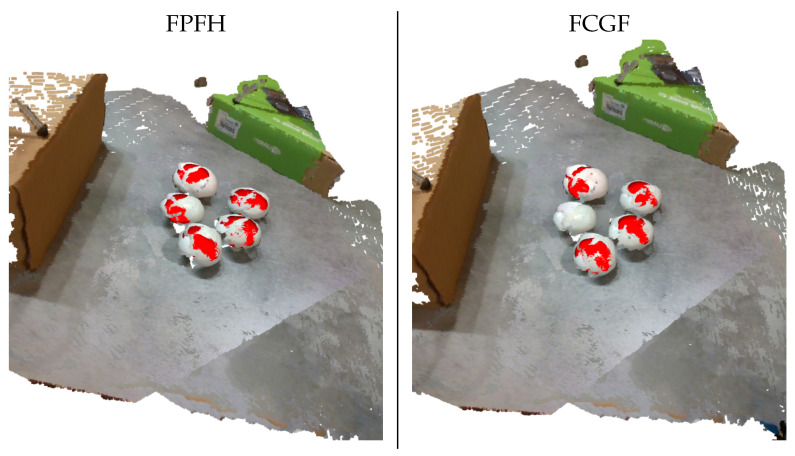
Visualization of the RANSAC-based estimation results for FPFH (first column) and FCGF (second column) features.

In Figure 9, we provide visual examples of the mushroom detection pipelines for both FPFH and FCGF options. Notably, the handcrafted FPFH features were more effective in the majority of cases. Specifically, deep FCGF features might miss some mushrooms entirely. Nevertheless, FCGF features were trained on an entirely different setting and, thus, fine-tuning the FCGF features by re-training the underlying Deep Neural Network (DNN) on synthetic mushroom data could be helpful. However, such a solution was outside of the scope of this work. For the rest of the paper, we considered FPFH features to be the default option.

## 6. Proposed Pipeline

The RANSAC-based algorithm worked well, considering its simplicity and the fact that it is a learning-free template matching approach. Nonetheless, there were considerable weaknesses:

1. We had to know an estimation of the minimum number of mushrooms to be searched if we wanted to detect them all, and this process could be rather slow.
2. It could be significantly inaccurate in some cases, especially considering that an off-the-shelf feature extractor was used and that several “valid” matches could occur due to the inherent symmetry of the mushroom cap.

**Figure 10:**
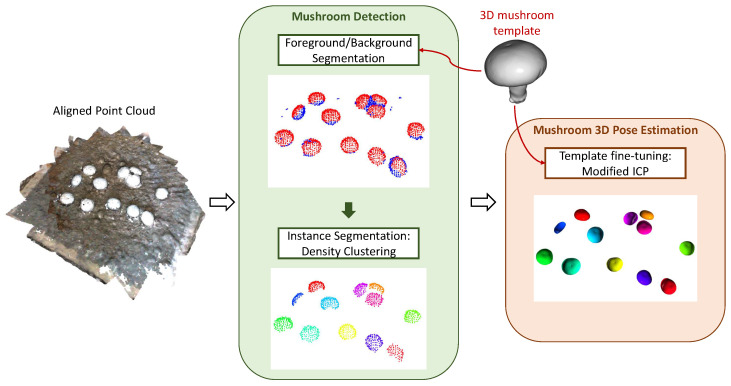
Overview of the proposed pipeline. The two main functionalities are mushroom detection and mushroom pose estimation. Visualization examples of each step/sub-step are provided.

To this end, we focused on introducing a mushroom segmentation step and a subsequent pose fine-tuning step. Specifically, this second template matching algorithm consisted of the following steps: (1) Mushroom Segmentation (Detection) and (2) Template Iterative Fine-Tuning (3D Pose Estimation). A high-level overview of this pipeline and its key components is shown in Figure 10.

### 6.1. Mushroom Instance Segmentation

For this step, we introduced an approach akin to one-class classification, where we assumed that all the mushroom points corresponded to 3D features of specific patterns. As 3D features, we could use the L2-normalized versions of either the FPFH or the FCGF features described in the previous section. To learn these mushroom-specific patterns, we extracted the 3D features of the template’s point cloud and then fitted a model on these data. We explored various ideas for this model fitting step, including one-class support vector machines, k-means, and Gaussian mixture models. Nonetheless, the best results, in practice, were produced when using a k-Medoids approach. This was not surprising, since the extracted features are normalized and, thus, lie on a hyper-sphere. As a result, mean-based approaches produce centers that may not belong to the manifold of the features. On the other hand, the use of k-Medoids selected, as the center of a cluster, one of the actual feature vectors. In this way, we had easily interpretable distance metrics between the center of the clusters and new features. Therefore, given the k-Medoids model, we decided whether a point from the actual point cloud scene belonged to a mushroom cap, or not, by means of a user-defined distance threshold with respect to distance to any of the cluster centers.

Two user-defined parameters control the k-Medoids approach: (1) the number of clusters and (2) the distance threshold. The number of clusters equals the number of “distinct” patterns we want to recognize. This number was set to 15, a fairly high number given the symmetry of the mushroom. We experimented with this parameter and no notable changes were reported when we avoided a very limited number of centers (e.g., <5). Computing distances over this model of normalized features is equivalent to cosine distance. Thus, we could set intuitive distance thresholds that corresponded to high similarity scores. We set the distance threshold to 0.2, or equivalent to 0.8 cosine similarity. This value provided a constrained neighborhood of possible similar results. As expected, the number of centers could slightly alter the appropriate distance threshold; more clusters required a lower threshold, while fewer clusters required a more loose threshold.

**Figure 11:**
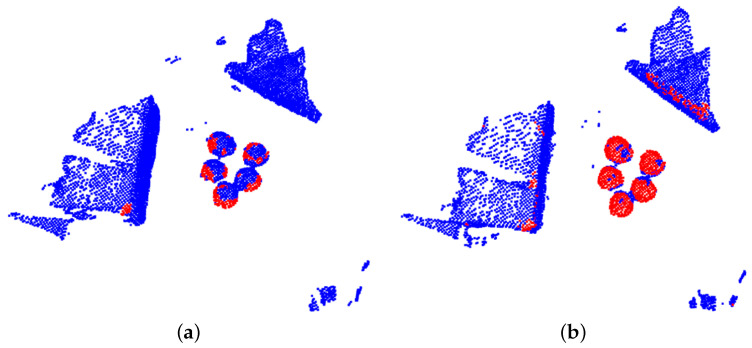
Examples of mushroom segmentation; points belonging to a mushroom are depicted with red color, while background points with blue. Two variants are depicted: (**a**) only one template point cloud is considered and (**b**) a set of augmented template point clouds are considered.

Note that, if we only used a simple instantiation of the template mesh, after randomly (uniformly) sampling points, and down-sampled, according to a predefined voxel-size, and then obtained the per-point 3D features, the proposed approach could not provide accurate results, as shown in Figure 11a. This was easily addressed by creating a set of augmented template versions. This augmentation process was performed according to the following steps:

- Randomly sample a different point cloud from the initial template mesh
- Use a modified voxel size by multiplying the initial user-defined voxel size with a value uniformly sampled from the range [0.8,1.2].
- Perform a 3D affine transformation using a constrained set of possible 3D rotations, as well as a constrained set of possible scales.
- Perform a local deformation of the mushroom surface along the surface normals. This step does not significantly affect the shape of the mushroom; it only helps to avoid “overfitting” of the model to very specific surface patterns.

Using the aforementioned augmentation scheme, we created 100 point cloud versions of the initial template, and then we used these point clouds to train our k-Medoid model. The difference in quality is evident in Figure 11b. Contrary to the single template case of Figure 11a, the augmented version managed to capture the majority of mushroom pixels. The slight increase in false positives was of no interest for the task at hand if the mushrooms were correctly segmented, since the upcoming steps could address falsely detected regions that did not resemble a mushroom shape. Moreover, the vision system should be applied to a mushroom bed in order to scan part of the mushroom cultivation where such distractors do not exist.

**Figure 12:**
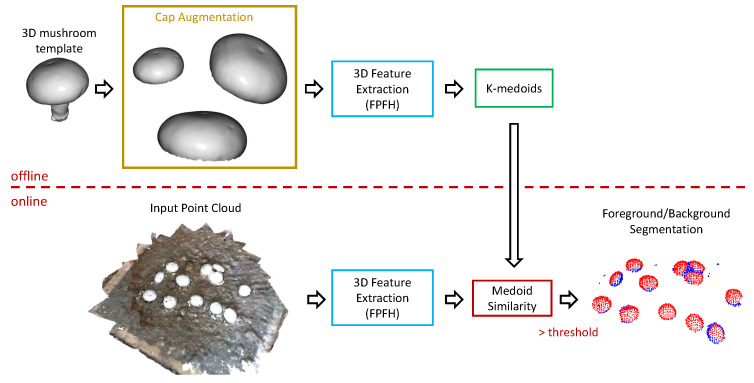
Overview of foreground/background separation. Augmentation of the template (only the cap), and clustering of the 3D features of the augmented set with a k-medoids algorithm, was applied offline. For the “online” processing of a new point cloud we simply compared the 3D feature of each point with the already computed medoids. If cosine similarity was above a user-defined threshold, the point was considered a mushroom point (i.e., foreground point).

The overview of the foreground/background segmentation system, which detects points of the point cloud that belong to mushrooms, is summarized in Figure 12, where we depict all the important components and distinguish the offline part of learning the medoids (that act as feature templates) and the online part of applying the segmentation pipeline to a new input point cloud.

**Figure 13:**
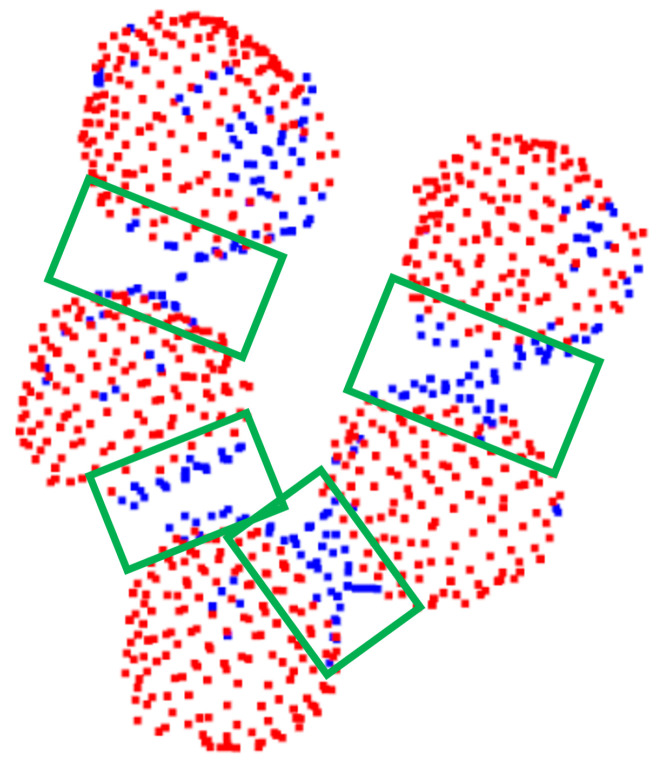
Example of foreground/background separation of neighboring mushrooms. Note that regions of “contact”, denoted with green boxes, were not recognized as mushrooms. Red points are classified as mushroom points (foreground) while blue points are not relevant points (background).

Thus far, we explored foreground/background segmentation in order to detect points of the point cloud that belong to actual mushrooms. Note that no other information, apart from 3D shape descriptions, in the form of 3D features, was used for this segmentation step. Using color information could be beneficial to filter the detected points further, since the case of interest, a specific mushroom type, has a white color. Different mushrooms should be separated into distinct labels to provide an instance segmentation solution. As we can see in Figure 11b, the proposed segmentation had an important desired property: regions of mushrooms that were close to each other were not recognized as mushrooms, as is further highlighted in Figure 13.

**Figure 14:**
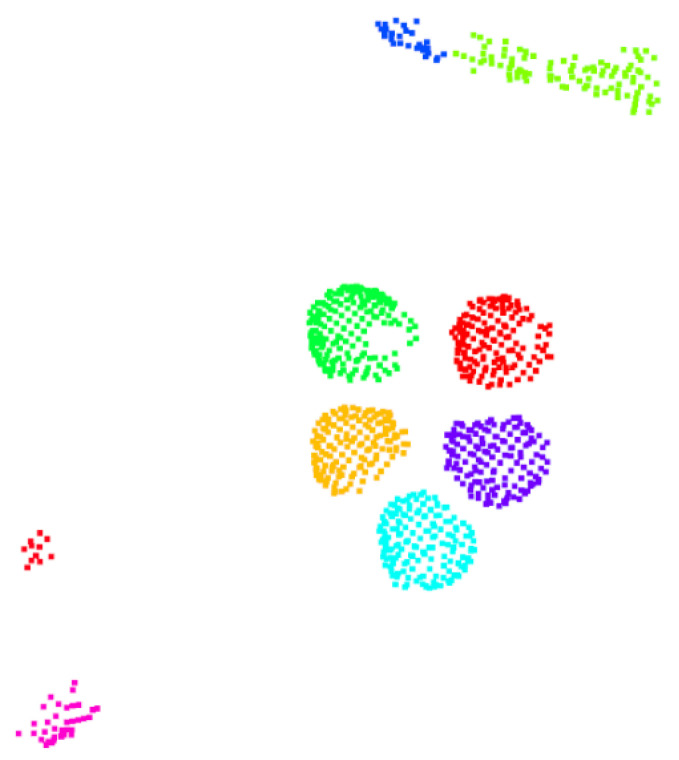
Visualization of the density clustering step over the foreground/background separation results. Different colors denote different clusters.

The aforementioned property enabled a density clustering solution, since we expected points of the same mushroom to be close enough and correctly predicted as mushroom parts. Thus, a clustering algorithm over the mushroom-labeled points was performed. Specifically, we applied the Density-based spatial clustering of applications with noise (Density-Based Spatial Clustering of Applications with Noise—DBSCAN [30]) algorithm. Since the number of mushrooms was unknown, such a density-based clustering approach was ideal, while it also removed potential noisy points. The clustering threshold, as requested by the DBSCAN algorithm, was intuitively set to 2× voxel-size. This step provided accurate instance segmentation results, as shown in Figure 14.

### 6.2. Pose Estimation via Template Iterative Fine-Tuning

Mushroom instance segmentation provides a 3D localization of individual mushrooms. Nonetheless, the problem of pose estimation has not been addressed. To this end, we developed an ICP-inspired approach. Specifically, given a well-defined mushroom cap region with a non-trivial subset of its corresponding 3D points, we performed an iterative approach to align the template point cloud to the segmented part as much as possible. Implementation-wise, we used a weighted ICP variant:

(1) $$E\left(M\right)=\sum_{(p,t)\in C}w\left(f\left(p\right),f\left(t\right)\right){\left(p-Mt\right)}^{2},$$

where *p*, *t* are points from the two point sets according to a correspondence set *C*, M is a similarity transform, f() is the feature extractor of the respective point and w() is the weight function given two feature vector. The latter weight function was the cosine similarity in our case. Here, correspondences *C* were not created by computing a single nearest neighbor, but we retrieved k nearest neighbors and then selected the neighbor with the highest feature similarity (in our case, we used k=3). This selection of more than one neighbor helped the convergence of the iterative process for our task in an intuitive manner: it helped us take “larger” steps towards good performing optima.

**Figure 15:**
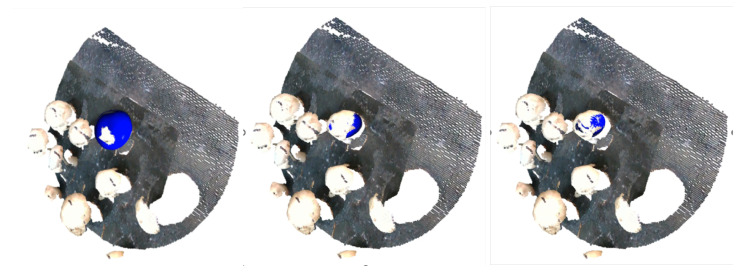
Visualization of three distinct (starting, intermediate and ending) steps of the developed ICP variant.

Distinct steps of this process are visualized in the Figure 15, where the fitted template mesh is visualized in a starting, an intermediate and an ending timestep.

**Figure 16:**
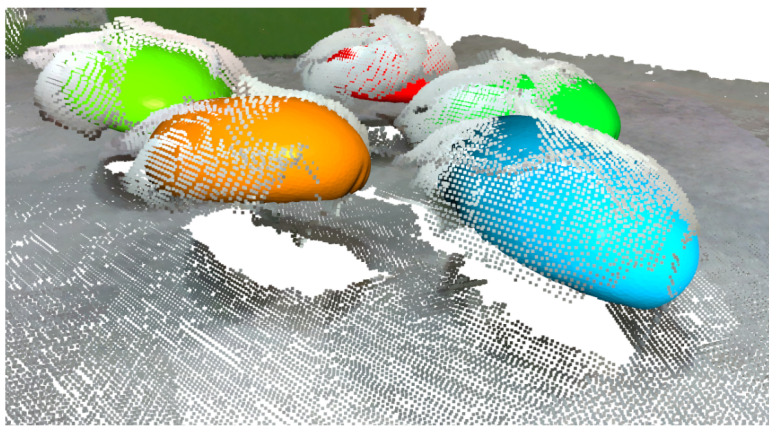
Detected mushrooms as template meshes of different colors.

**Figure 17:**
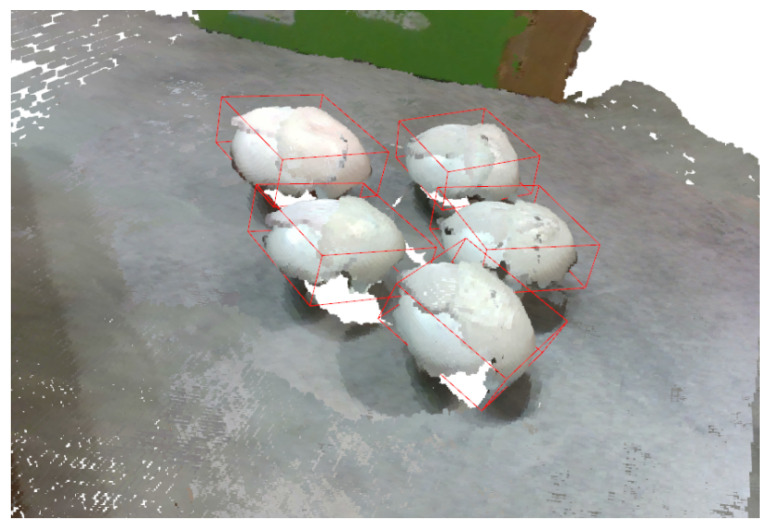
Mushroom pose estimation results in the form of oriented bounding boxes.

Naturally, to estimate the pose of all the mushrooms, this iterative method should be applied over all segmented regions of the previous instance segmentation step. Examples of pose estimation using the overall proposed pipeline are depicted in Figure 16, where we superimposed the detected transformed templates into the point cloud, and in Figure 17, where we denoted the detection with an oriented 3D bounding box.

**An Ellipsoid Alternative:** The mushroom cap has a very specific shape that resembles a 3D ellipsoid. Therefore, one might avoid the template matching scheme, which requires a matching step between points of two different point clouds, and straightforwardly apply a much faster ellipsoid fitting procedure. The resulting translation, scale and rotation of the ellipsoid would correspond to the respective transformation parameters of the template matching approach. To avoid outliers contributing to the fitting process, we followed a re-weighted iterative least-square approach as Equation (2) suggests, where we iteratively estimated $M\in R^{3\times 3}$ and $H\in R^{1\times 3}$ and then used these estimations to update the weight $w_{i}$. Specifically, Equation (2) describes a (normalized) general formulation of an ellipsoid, where M is a positive definite matrix.

(2) $$\sum_{i}w_{i}\left(x_{i}^{⊺}Mx_{i}+Hx_{i}+1\right)=0$$

Through these two matrices, (M,H), the ellipsoid and the ellipsoid parameters can be straightforwardly computed (e.g., the rotation matrix corresponds to the eigenvectors of M). The weights $w_{i}$ were updated as in Equation (3) in a typical re-weighted rationale.

(3) $$w_{i}=1/max\left(10^{-5},x_{i}^{⊺}Mx_{i}+Hx_{i}+1\right)$$

**Figure 18:**
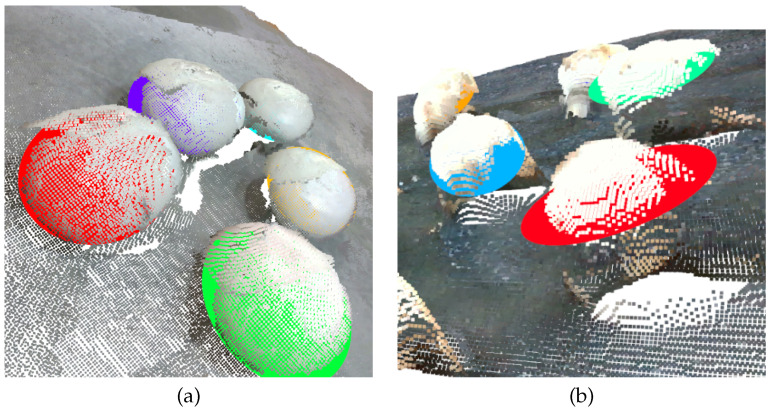
Examples of (**a**) successful and (**b**) erroneous ellipsoid fitting.

Even though, in some cases, this worked notably well (see Figure 18a), the partial information available from point clouds introduced ambiguity of the possible ellipsoid configurations and could result in erroneous pose estimation, as shown in Figure 18b. To this end, we followed the aforementioned template matching scheme, but we considered this ellipsoid-based approach promising and a constraint-based alternative will be explored in future work.

## 7. Experimental Evaluation

The major challenge of this work was the lack of annotation, originating from the inherent difficulty of annotating 3D objects, especially in cluttered environments. To this end, we created a synthetic dataset of mushroom scenes. In order to obtain a quantitative measure of the effectiveness of our methods, we evaluated them in this synthetic dataset. Then, we proceeded with qualitative results of visual examples in real data acquired from multiple RGBD cameras.

### 7.1. Synthetic Point Clouds

To create a synthetic dataset of mushroom scenes, we developed a mushroom scene generator that places mushrooms under different transformations, which results in realistic settings into a ground plane. The following steps summarize the whole scene generation process:

1 Use a realistic non-smooth ground plane. For this, a ground dirt mesh was used. For each scene, small local deformations were applied to the ground mesh.
2 Select a number *K* of mushrooms to be placed on the ground mesh. The whole mushroom template of Figure 3 was used in this step and not only the cap. For each mushroom to be placed, follow these transformation sub-steps:
  - Translate a mushroom template anywhere in the domain (over the *xy*-axes) defined by the ground mesh.
  - Apply a scale factor within the range [1−1/3,1+1/3]. Then, a finer per-axis re-scaling step is performed in a constrained range of [0.9,1.1], providing extra variability in the shape of the mushroom. The mushroom is then translated along the *z*-axis in order to have its bottom point in the proximity of the ground plane.
  - Apply a 3D rotation according to randomly selected axis angles. Rotation over the *x*- and *y*-axes was constrained to the range $\left[-30^{\circ },30^{\circ }\right]$. Due to symmetry over the *z*-axis, we chose to leave this rotation unconstrained.
  - Apply local deformations in the mushroom mesh (along the surface normals) without significantly altering its surface. Implementation-wise, a small number of vertices were randomly selected to be translated along their corresponding normal. Small values were considered in order to retain the surface shape. Neighboring vertices were also deformed following an interpolation rationale.
  - Apply basic collision checking. If a newly created mushroom collided with an existing one, we discarded the new mushroom and created another new one until no collision was detected.
3 Create the final point cloud, using a slightly different sampling number of points and different voxel sizes (×0.8–×1.2) for the subsequent down-sampling in order to provide extra variability in the created scenes.
4 Lastly, simulate realistic collected point clouds, for which a hidden point removal step was performed [31]. This step approximated the visibility of a point cloud from a given view and removed the occluded points. Possible views were randomly selected using a constrained radius range and a constrained set of axis angles that did not considerably diverge from an overhead viewpoint.

**Figure 19:**
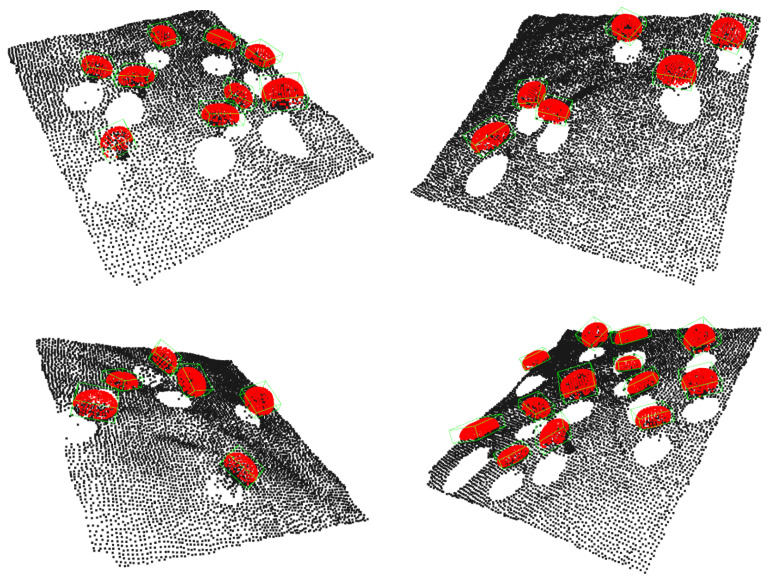
Examples of synthetic mushroom scenes. Mushroom caps are highlighted with red color.

Examples of the generated mushroom scenes can be found in Figure 19.

### 7.2. Quantitative Evaluation

In this section, we provide ablation studies over various modifications discussed throughout the manuscript. Evaluation was performed over the synthetic mushroom scenes. Specifically, we created a dataset of 50 mushroom scenes and ∼500 mushrooms in total. Each scene had a random number of mushrooms between 5 and 15. Before measuring pose estimation accuracy, we found the correspondences between the existing mushrooms and their potential predicted candidates. Thus, our initial evaluation was performed through retrieval metrics. Mean Average Precision (MAP) was reported, with an overlap Intersection-over-Union (IoU) threshold dictating successful detection. Since the two sub-tasks of detection and pose estimation intervened, such a retrieval-based take on the problem could provide a quantitative evaluation. Each experiment was repeated five times, and mean values reported.

#### 7.2.1. RANSAC-Based vs. Proposed Pipeline

We start by highlighting the proposed pipeline’s effectiveness, compared to the RANSAC approach of Section 5. Mushroom retrieval results for two different overlap thresholds (25% and 50% IoU) are presented in Table 1. It is evident that the proposed pipeline outperformed the basic template matching pipeline of Section 5, retaining very high retrieval scores, even when using a 50% IoU threshold. This behavior implied that the RANSAC-based method found the position of most of the mushrooms (high score at 25% IoU—not as high as the proposed pipeline), but could not accurately perceive the pose (reduced score at 50% IoU).

#### 7.2.2. Ablation over Implementation Choices

Here, we go through the different modifications introduced in the proposed method, as described in Section 6. In Table 2, we compare the retrieval results of the proposed pipeline when using different 3D features. Specifically, we used FPFH [27] and FCGF [28] features (for more details, see Section 5). We can see that the pre-trained off-the-shelf FCGF features underperformed for the 50% IoU threshold, even though their performance was almost perfect for a lower IoU threshold. Again, this is an indication of erroneous pose estimation.

In Table 3, a similar performance drop for 50% IoU was observed for a typical ICP fine-tuning step instead of the proposed modification of Section 6. These results highlighted the necessity of the modified ICP variant.

We also experimented with the optional step of surface reconstruction, as described in Section 4. According to the results of Table 4, using the surface reconstruction step led to similar performance, whilst larger standard deviation was observed. When considering the computational overhead of this extra step, we could safely deduce that this step was redundant application-wise.

Finally, we also evaluated the performance of the ellipsoid alternative, described at the end of Section 6. The results are reported in Table 5. As expected, using the ellipsoid variant as the template matching step yielded subpar performance, compared to the modified ICP. Nonetheless, the reported performance was close to 90% despite its simplicity. This result was a promising indication of the effectiveness of this lightweight alternative, especially if constraints on the scale/rotation of the ellipsoid are added.

#### 7.2.3. Quantify Pose Estimation

**Figure 20:**
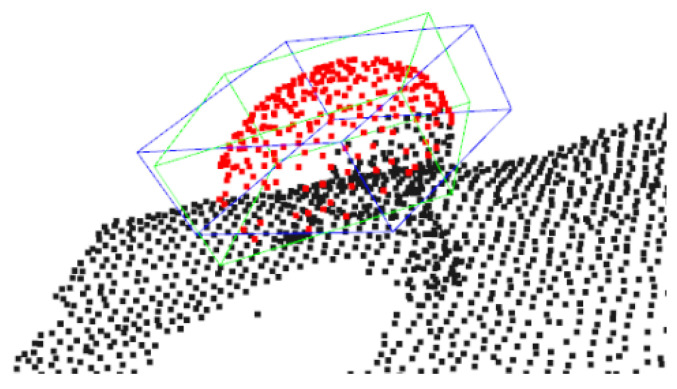
Two bounding boxes of very similar pose that report an overlap IoU value of 60%. The green box denotes the ground truth box, while the blue box denotes the predicted bounding box.

Retrieval metrics served as useful indications of both the detection and the pose estimation of mushrooms. Nonetheless, trying to use a high IoU (>66%) threshold led to poor performance. In theory, higher IoU should narrow down the mushroom set to a subset of very accurately estimated poses. Nonetheless, an inherent problem of oriented bounding boxes arises in such settings. Due to the symmetry of the mushroom, orientation around the *z*-axis (of the initial upright template) should not affect the metric of pose estimation. This was not the case for bounding boxes, as shown in Figure 20, since orientation around the *z*-axis could create non-trivial fluctuations over the IoU overlap ratio.

A more in-depth evaluation of the pose estimation was performed through the dot product of the normals, as calculated at the top surface of the extracted bounding boxes. This was equivalent to the cosine similarity of the corresponding rotation vectors. In Table 6, we report the average and the median cosine similarity for every mushroom detection over the respective IoU threshold. As expected, the reported cosine similarity values increased for increased IoU thresholds since fewer, as more similar detection was considered. Nevertheless, cosine values were very high in all reported cases, highlighting the correlation between the ground truth and the detected pose. Table 6 also contains mean/median angle errors to provide an intuitive metric concerning pose estimation.

#### 7.2.4. Comparison to Existing Methods

As we mentioned in Section 2, we only identified the work of Baisa et al. [5] as a closely related approach that deals with the same problem of mushroom pose estimation. We should highlight that the detection step of their approach relied on circle detection (via Hough transform), which can produce reliable results only when an overhead view is considered and without intense cap angles. In fact, when the view is changed or the mushroom is not upright, the most consistent shape to approximate the image projection of the 3D mushroom caps is an ellipse. We consider our segmentation approach of great significance, as it alleviates several assumptions (e.g., camera view) and provides notable results, even when the mushrooms touch each other. Nonetheless, due to the differences in the input of this step, we could not simulate the exact pipeline of [5] (evaluation was performed only on synthetic point cloud data, where 3D annotations were available, while the segmentation step [5] assumed images of an overhead view as input). Nonetheless, we could compare the pose estimation component of [5], given a well-cropped point cloud region of each mushroom. Note that the mushroom region provided by our segmentation step typically did not contain points of neighboring mushrooms, which was not necessarily the case for the segmentation step of [5]. In more detail, the pose estimation step of [5] relies on a typical registration pipeline consisting of two steps: (1) RANSAC for global registration and (2) ICP for a finer alignment of the template. In other words, in the context of our work, we could simulate this pose estimation pipeline by applying the explored RANSAC-based template matching with segmented mushroom scenes. The results of this approach, compared to ours, is presented in Table 7.

As we can observe from the results, even when segmentation was very good, the upcoming registration step might considerably deteriorate performance for a pipeline similar to [5]. This was in line with the following observations so far made: (1) RANSAC-based approaches on such a symmetric object may lead to much erroneous matching that further increases the difficulty of the problem (see also Table 1, even when only a region of a single mushrooms is considered; (2) the ICP approach is very sensitive (see also Table 3) and, especially for this case, poor initialization from the RANSAC global registration could further deteriorate performance when using ICP.

### 7.3. Qualitative Evaluation

**Figure 21:**
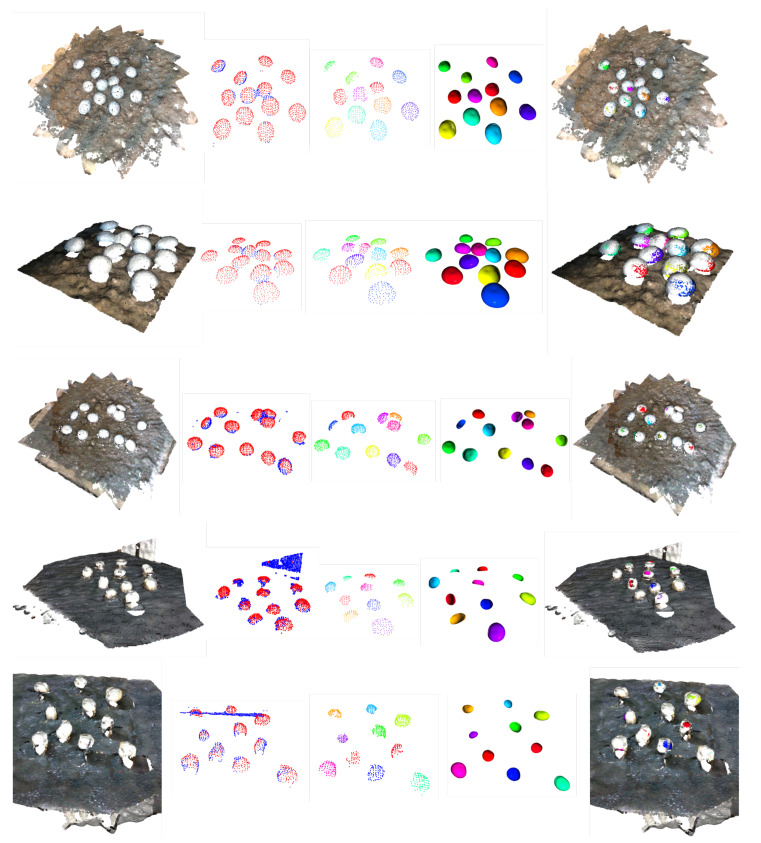
Visualization of the proposed steps when real data were considered. From left to right: (1) initial point cloud, (2) foreground/background separation, (3) instance segmentation, (4) template matching, (5) fitted templates overlaid over the initial pointcloud. The first three rows correspond to point cloud from multiple viewpoints of a rotating vision system, while the last two correspond to point clouds of two opposite views.

Having explored the effectiveness of the proposed method over the synthetic mushroom dataset, we proceed by providing qualitative results in real mushroom scenes captured by the discussed multi-camera settings of Section 3. Specifically, we provide visualization examples of the basic steps of the proposed pipeline for both the multi-view setting of 3D printed mushrooms, as shown in the first three rows of Figure 21, and the two-view setting of real mushrooms, as shown in the last two rows of Figure 21. As we can see, the proposed approach detected all the mushrooms existing in the scenes and re-created their poses with the visualized templates to a notable level. We should highlight how well the proposed approach worked, even when the mushrooms were very close to each other, as shown in the second and third rows of Figure 21.

## 8. Conclusions

In this work, we explored the task of mushroom detection and mushroom 3D pose estimation in an annotation-free context. The developed approaches rely only on a mushroom template in the form of a 3D mesh/point cloud. To tackle the 3D pose estimation, information on the 3D structure of the scene is important. To this end, we focused on data acquired from RGB-D sensors and processed as point clouds. To further assist the understanding of the scene, we considered multiple sensors/views. The proposed approach uses the mushroom template to separate points belonging to mushrooms from their backgrounds in a one-class classification rationale. A density clustering algorithm follows this step to provide an instance segmentation of the different mushrooms. Then, each mushroom region is approximated with the mushroom template using a modified ICP algorithm. An ellipsoid alternative was also explored as a faster alternative. The developed methods were evaluated over a synthetic dataset of mushroom scenes, as well as over a set of real collected data of multi-view point clouds. The proposed approach was effective in all considered settings, despite its simplicity. Creating the synthetic dataset and using pre-trained deep 3D features pave the way toward future research endeavors. One could fine-tune 3D deep learning models on the synthetic mushroom scenes and evaluate their performance on real data.

## Figures and Tables

**Table 1:** Comparison of the RANSAC-based approach and the proposed pipeline.

Method	MAP @ 25% IoU	MAP @ 50% IoU
RANSAC-based Approach	90.89%	53.62%
Proposed Approach	99.80%	96.31%

**Table 2:** Impact of different 3D features. FPFH [27] and FCGF [28] were considered.

3D Features	MAP @ 25% IoU	MAP @ 50% IoU
FCGF	99.63%	24.75%
FPFH	99.80%	96.31%

**Table 3:** Impact of the ICP template fine-tuning step. Typical ICP and the proposed modification are compared.

Template Alignment	MAP @ 25% IoU	MAP @ 50% IoU
basic ICP	98.33%	45.92%
modified ICP	99.80%	96.31%

**Table 4:** Impact of the optional mesh reconstruction preprocessing step.

	w/Surface Reconstruction	w/o Surface Reconstruction
MAP @ 50% IoU:	96.36 ± 2.02%	96.31 ± 0.92%

**Table 5:** Comparison between the ellipsoid-based pose estimation and the proposed template matching ICP variant.

	Ellipsoid Variant	Modified ICP
MAP @ 50% IoU:	89.09%	96.31%

**Table 6:** Pose Estimation in terms of cosine similarity between rotation vectors. Mean and median cosine metrics were computed for detection over a specified IoU threshold.

	25% IoU	50% IoU
mean cos. similarity:	0.9885	0.9920
mean angle error:	8.70${}^{\circ }$	7.27${}^{\circ }$
median cos. similarity:	0.9927	0.9948
median angle error:	6.93${}^{\circ }$	5.85${}^{\circ }$

**Table 7:** Detection and pose estimation results for a standard registration pipeline (RANSAC+ICP) applied on segmented mushrooms regions. We report both MAP and angle error for detections over 25% IoU.

	MAP	(Mean) Angle Error
segmented RANSAC+ICP	91.78%	13.77${}^{\circ }$
proposed	99.80%	8.70${}^{\circ }$

## Data Availability

Point clouds of real data are limited and will be available at the end of the SoftGrip project. Synthetic data are created through a script using the available mushroom template mesh. To obtain the scene creation script, contact the authors.
